# *Mycobacterium tuberculosis* Thioredoxin Reductase Is Essential for Thiol Redox Homeostasis but Plays a Minor Role in Antioxidant Defense

**DOI:** 10.1371/journal.ppat.1005675

**Published:** 2016-06-01

**Authors:** Kan Lin, Kathryn M. O'Brien, Carolina Trujillo, Ruojun Wang, Joshua B. Wallach, Dirk Schnappinger, Sabine Ehrt

**Affiliations:** 1 Department of Microbiology and Immunology, Weill Cornell Medical College, New York, New York, United States of America; 2 Program in Immunology and Microbial Pathogenesis, Weill Graduate School of Medical Sciences of Cornell University, New York, New York, United States of America; National Institutes of Health, UNITED STATES

## Abstract

*Mycobacterium tuberculosis* (*Mtb*) must cope with exogenous oxidative stress imposed by the host. Unlike other antioxidant enzymes, *Mtb’s* thioredoxin reductase TrxB2 has been predicted to be essential not only to fight host defenses but also for *in vitro* growth. However, the specific physiological role of TrxB2 and its importance for *Mtb* pathogenesis remain undefined. Here we show that genetic inactivation of thioredoxin reductase perturbed several growth-essential processes, including sulfur and DNA metabolism and rapidly killed and lysed *Mtb*. Death was due to cidal thiol-specific oxidizing stress and prevented by a disulfide reductant. In contrast, thioredoxin reductase deficiency did not significantly increase susceptibility to oxidative and nitrosative stress. *In vivo* targeting TrxB2 eradicated *Mtb* during both acute and chronic phases of mouse infection. Deliberately leaky knockdown mutants identified the specificity of TrxB2 inhibitors and showed that partial inactivation of TrxB2 increased *Mtb’s* susceptibility to rifampicin. These studies reveal TrxB2 as essential thiol-reducing enzyme in *Mtb in vitro* and during infection, establish the value of targeting TrxB2, and provide tools to accelerate the development of TrxB2 inhibitors.

## Author Summary

*Mycobacterium tuberculosis* (*Mtb*) antioxidant systems represent attractive targets for developing novel tuberculosis therapies. We demonstrate that targeting thioredoxin reductase TrxB2 eradicates *Mtb* during acute and chronic mouse infections. TrxB2 inactivation caused thiol-specific oxidizing stress, perturbed growth-essential processes and resulted in lytic death. Unexpectedly, TrxB2 deficiency did not cause increased susceptibility to oxidative and nitrosative stress. To uncover the mechanistic consequences of depleting TrxB2, or other growth essential proteins, in viable and growing bacteria, we developed a “leaky” knockdown system, with which partial TrxB2 depletion was achieved. Importantly, these leaky mutants revealed that one of two TrxB2 inhibitors kills *Mtb* via TrxB2 inactivation. They also demonstrated that TrxB2 depletion results in hypersusceptibility to rifampicin suggesting that a TrxB2 inhibitor will synergize with this frontline anti tuberculosis drug.

## Introduction

Endogenous oxidative stress represents an inevitable challenge for microbes adapted to an aerobic lifestyle [1]. In addition, pathogens like *Mycobacterium tuberculosis* (*Mtb*) are confronted with exogenous oxidative stress imposed by the host [2]. The production of antimicrobial oxidants is a critical host defense mechanism against *Mtb* [3,4]. Patients with germline mutations in phagocyte NADPH oxidase resulting in an impaired macrophage respiratory burst are predisposed to mycobacterial diseases including tuberculosis [5]. Mice lacking inducible nitric oxide synthase succumb to *Mtb* infection much faster than their wild type littermates [3]. The reactive oxygen and nitrogen species generated by these host enzymes can inactivate microbial iron-dependent enzymes, damage lipids and destroy DNA [1,6].

Not unexpectedly, *Mtb* is armed with a number of dedicated antioxidant systems to ensure replication and survival within its host. Notable members include catalase, alkyl hydroperoxidase, superoxide dismutase, mycothiol, ergothioneine, thiol peroxidase, thioredoxin reductase and a recently identified membrane-associated oxidoreductase complex [4,7–13]. The thioredoxin system, together with the glutathione system, regulates many important cellular processes, such as antioxidant pathways, DNA and protein repair enzymes, and the activation of redox-sensitive transcription factors [6,14]. Unlike many Gram-negative bacteria, which possess both systems, *Mtb* lacks the glutathione system [6,10]. Instead, mycothiol has been suggested as substitute for glutathione in *Mtb* [10]. Mycothiol-deficient *Mtb* requires addition of catalase for growth *in vitro*, but is not significantly attenuated in mice [15]. In contrast, there is evidence that thioredoxin reductase (TrxB2) is essential for growth *in vitro*, implying a unique role for TrxB2 [16–18]. Although purified TrxB2 has been shown to mediate detoxification of H_2_O_2_, peroxide, and dinitrobenzene *in vitro* [12,19,20], its role in oxidative stress defense in physiological conditions and its specific biological functions in *Mtb* physiology are poorly understood. Bacterial thioredoxin reductases have recently been demonstrated to be druggable targets [18,21], however, it has not been determined whether inactivating TrxB2 *in vivo*, in acute and chronic infections, attenuates *Mtb*.

To address these questions, we applied a tunable dual-control genetic switch [22] to generate a conditional TrxB2 mutant and evaluated the impact of TrxB2 depletion. Unexpectedly, depleting TrxB2 not only rapidly killed *Mtb*, but also led to bacterial lysis. TrxB2 depletion perturbed growth-essential processes, including sulfur and DNA metabolism and death could be prevented by addition of a strong disulfide reductant. *In vivo* depletion of TrxB2 resulted in clearance of *Mtb* during both the acute and chronic phases of infection. We generated deliberately leaky knockdown mutants to dissect the contribution of TrxB2 to oxidative stress detoxification and found *Mtb* with partially depleted TrxB2 highly susceptible to thiol-specific oxidizing stress, but, surprisingly, not to peroxide and reactive nitrogen species. The leaky knockdown mutants were used to evaluate the specificity of two TrxB2 inhibitors and revealed that targeting TrxB2 results in hypersusceptibility to the frontline anti-tuberculosis drug rifampicin.

## Results

### TrxB2 is essential for growth and survival of *Mtb in vitro*

**Fig 1:**
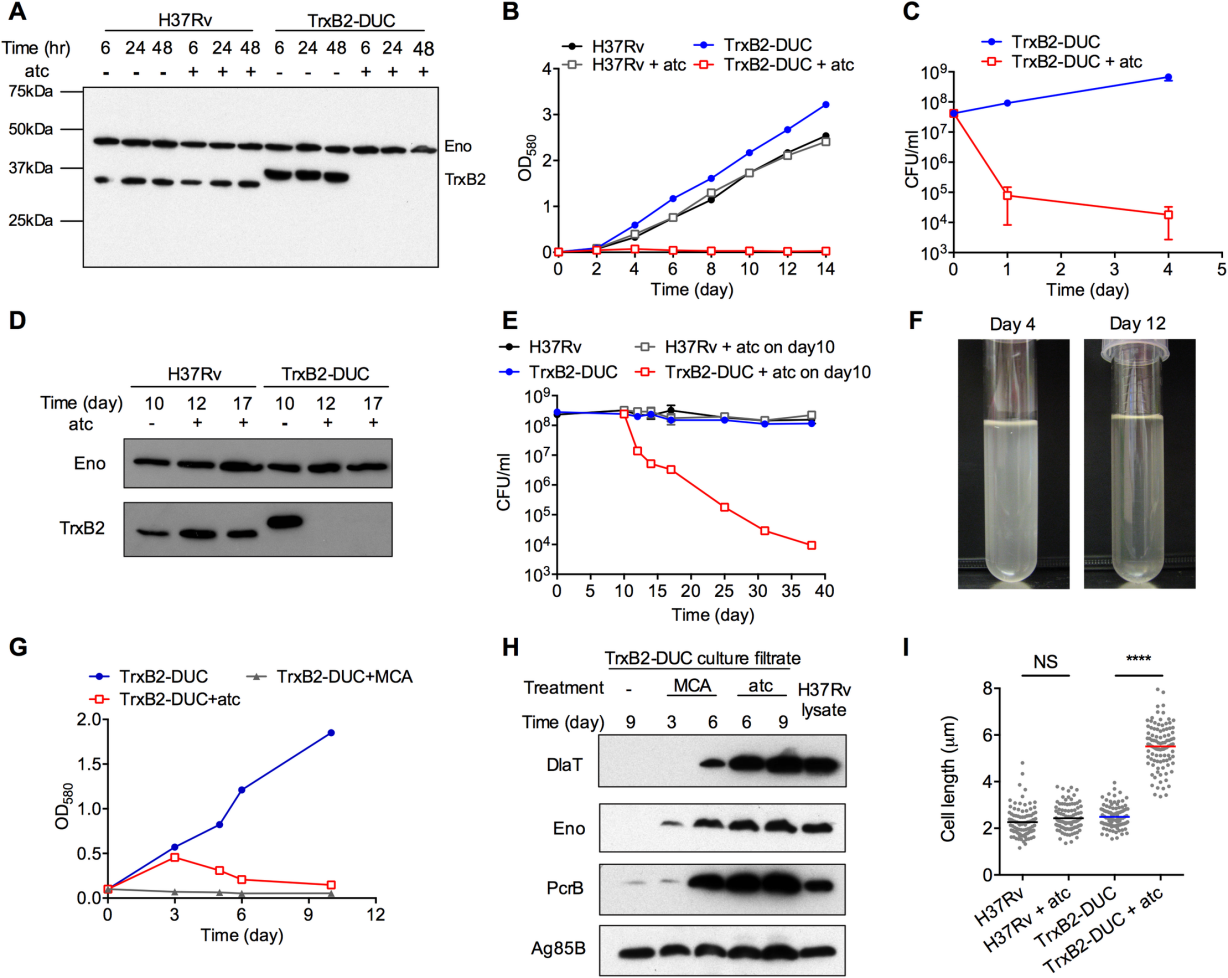
TrxB2 is essential for *Mtb* survival in replicating and non-replicating conditions. (A-C) Impact of TrxB2 depletion on replicating *Mtb*. (A) Immunoblot of protein extracts from H37Rv and TrxB2-DUC grown with and without atc. Blot was probed with TrxB2-specific and Eno-specific (loading control) antisera. TrxB2 in the TrxB2-DUC mutant is of increased molecular weight due to the C-terminal DAS tag. (B) Growth of individual *Mtb* strains quantified by optical density in nutrient-rich medium with or without atc. Starting density of the cultures was OD_580_ ~ 0.01. (C) Survival of *Mtb* strains quantified by CFU in 7H9 medium with or without atc (n = 6 per group). (D and E) Impact of TrxB2 depletion on non-replicating *Mtb*. (D) Immunoblot of protein extracts from *Mtb* cultures during starvation in PBS with or without atc. H37Rv and TrxB2-DUC were suspended in PBS for 10 days to obtain a non-replicating state. Where indicated, atc was added to the cultures on day 10. (E) Quantification of CFU from cultures in (D) at the indicated time points (n = 3 per group). (F) Appearance of the atc-treated TrxB2-DUC culture in 7H9 medium on day 4 and on day12. (G) Growth of TrxB2-DUC in 7H9 medium, treated with atc or meropenem–clavulanate (MCA). Starting density of the cultures was OD_580_ ~ 0.1. (H) Immunoblot analysis of dihydrolipoamide acyltransferase (DlaT), enolase (Eno), proteasome beta subunit (PrcB) and secreted protein antigen 85B (Ag85B) from culture supernatants in (G) at the indicated times. (I) Quantification of cell length by microscopy of indicated *Mtb* strains treated or not with atc for 4 days. Mean cell lengths (n = 100) are indicated. **** p<0.0001 by one-way ANOVA. All results are representative of at least three independent experiments. Data in (C) and (E) are means ± SD. In some panels, error bars are too small to be seen.

We first established that TrxB2 is indeed required for growth of *Mtb* under standard laboratory conditions (S1 Fig). Because a deletion mutant could not be isolated, we generated a TrxB2 dual-control (DUC) strain (S2 Fig). In TrxB2-DUC expression of TrxB2 is controlled by both transcriptional silencing and inducible proteolytic degradation, while TrxC is constitutively expressed from its native promoter [22]. Upon addition of anhydrotetracycline (atc) TrxB2 protein was rapidly depleted and below the limit of detection after 6 hours, which corresponds to less than 5% of TrxB2 amount in wild type (wt) H37Rv (Fig 1A and S3 Fig). TrxB2 depletion not only inhibited *Mtb* growth in nutrition-rich 7H9 medium, but also led to rapid killing (Fig 1B and 1C). Bacterial viability declined by 2.7 log after 24 hours, and 3.4 log after 4 days of atc treatment, indicating that TrxB2 is required for bacterial growth and survival in replicating conditions. We also assessed the impact of inactivating TrxB2 on non-replicating *Mtb*, which is known to be tolerant to anti-TB drugs and, in part, responsible for the long duration of anti-TB chemotherapy [23]. TrxB2 depletion was induced with atc after 10 days of incubation in PBS. Remarkably, TrxB2 depletion killed ~90% of the bacilli after 48 h and 99.9% within two weeks of PBS starvation, highlighting that starvation-induced non-replicating *Mtb* depends on TrxB2 for survival as well (Fig 1D and 1E).

### Depleting TrxB2 caused lytic death of *Mtb*

While culturing TrxB2-depleted *Mtb* in liquid growth medium, we observed that the culture gradually declined in optical density and turned clear (Fig 1F and 1G). This motivated us to ask whether TrxB2 depletion caused lysis of *Mtb*. Notably, mycobacterial death is not always accompanied by lysis. So far, only a small number of cell-wall targeting compounds have been shown to induce lytic death [24]. To further investigate whether lysis occurred upon TrxB2 depletion, we monitored the release of the cytoplasmic enzymes enolase (Eno), dihydrolipoamide acyltransferase (DlaT) and the proteasome beta subunit (PrcB) into the culture supernatant. Because Eno, DlaT and PrcB are generally not detected in the culture supernatant of intact mycobacterial cells, we consider their release as an indicator of bacterial lysis. Consistent with a previous report that meropenem-clavulanate caused *Mtb* lysis [24], we found Eno, DlaT and PrcB in the culture filtrate 6 days after exposure to meropenem-clavulanate (Fig 1H). There was no detectable lysis of TrxB2-DUC in the absence of antibiotic or atc, even after 9 days of incubation. In contrast, cytoplasmic proteins were readily detectable in the supernatant of TrxB2-DUC treated with atc for 6 or 9 days, confirming our hypothesis that TrxB2 depletion caused lytic death (Fig 1H). In contrast, depletion of nicotinamide adenine dinucleotide synthetase (NadE) which also rapidly kills *Mtb* [22], did not result in detectable lysis of NadE-DUC (S4 Fig). Microscopic analysis revealed that lysis of TrxB2-depleted *Mtb* was preceded by significant cell elongation (Fig 1I and S5 Fig). The majority of TrxB2-depleted bacteria were twice as long as those expressing TrxB2, suggesting that TrxB2 depletion affects processes required for cell division.

### TrxB2 is essential for *Mtb* to establish and maintain infection in mice

**Fig 2:**
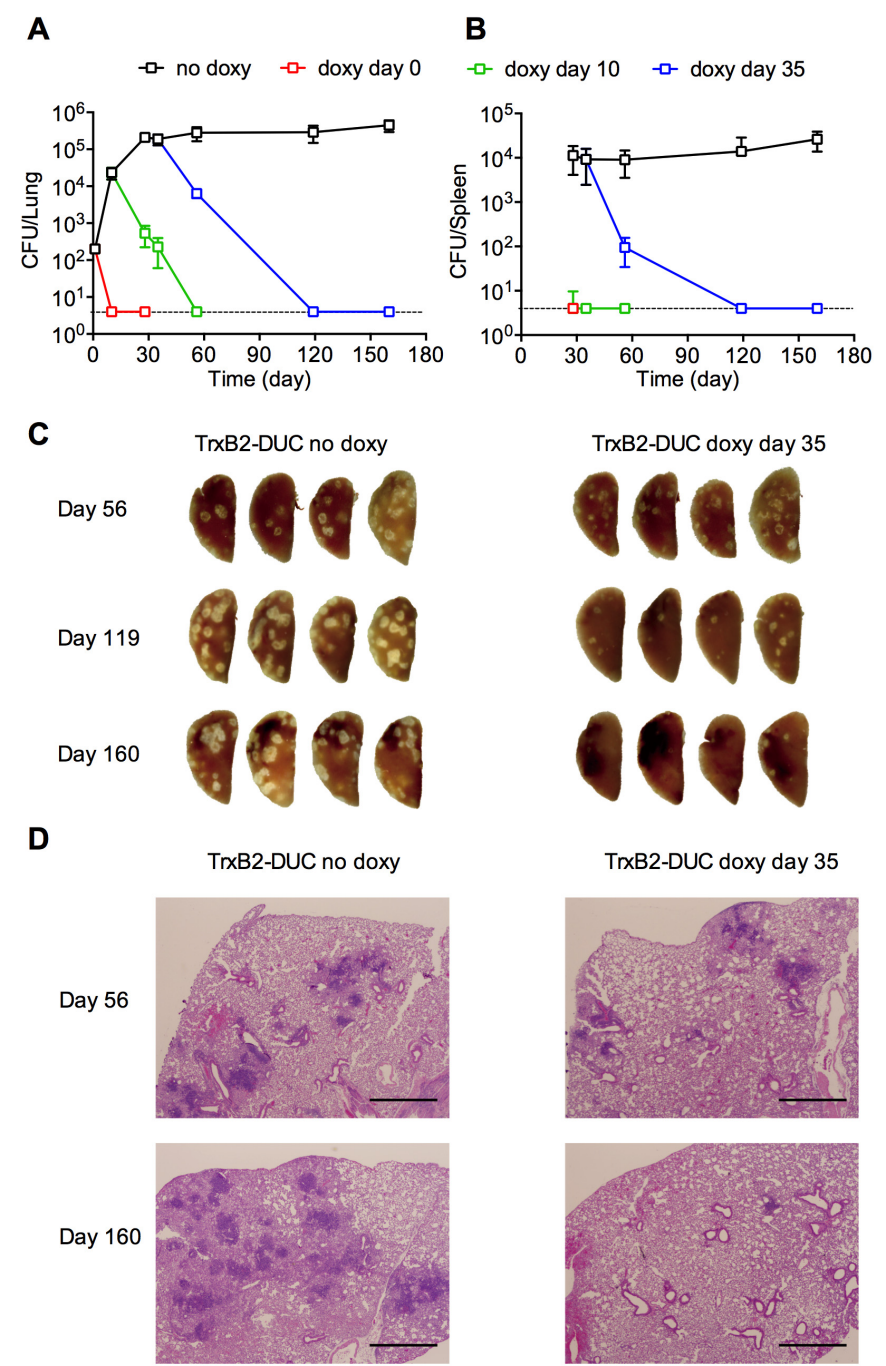
TrxB2 is essential for *Mtb* to establish and maintain infection in mice. (A and B) Quantification of bacterial loads in lungs (A) and spleens (B) of C56BL/6 mice infected with TrxB2-DUC. Mice received doxy-containing food starting at the indicated time points or not at all. Data are means ± SD of four mice per group. The limit of detection was 4 CFU per organ and is indicated by the dashed line. (C) Gross pathology of lungs from infected mice receiving doxy-containing food starting on day 35 or not at all. Lungs were isolated on day 56, 119 and 160 post infection. (D) Haematoxylin/eosin-stained lung tissue sections from mice infected with TrxB2-DUC not treated or treated with doxy-containing food starting on day 35 post infection. Images are representative of the histopathology of the four mice from each group. Scale bar, 1.0 mm. Results are representative of two independent experiments.

To evaluate the importance of TrxB2 for virulence of *Mtb*, mice were infected with TrxB2-DUC and fed doxycycline (doxy) containing food to inactivate TrxB2 at selected time points. The infection was rapidly cleared in mice given doxy food from the time of infection or during the acute phase of infection on day 10 (Fig 2A and 2B). No pulmonary pathology was observed in these mice (S6 Fig). Even when TrxB2 depletion was initiated during the chronic phase of infection on day 35, colony forming units (CFU) declined rapidly and no bacteria could be isolated from both lungs and spleens on day 160 (Fig 2A and 2B). The decline of CFU was accompanied by progressive healing of lesions in the lungs (Fig 2C and 2D). These results establish that TrxB2 is required for growth and persistence of *Mtb* in mice and point to the value of targeting TrxB2 to treat TB.

### Leaky TrxB2-TetON mutants reveal a specific role for TrxB2 in preventing thiol-oxidizing stress

**Fig 3:**
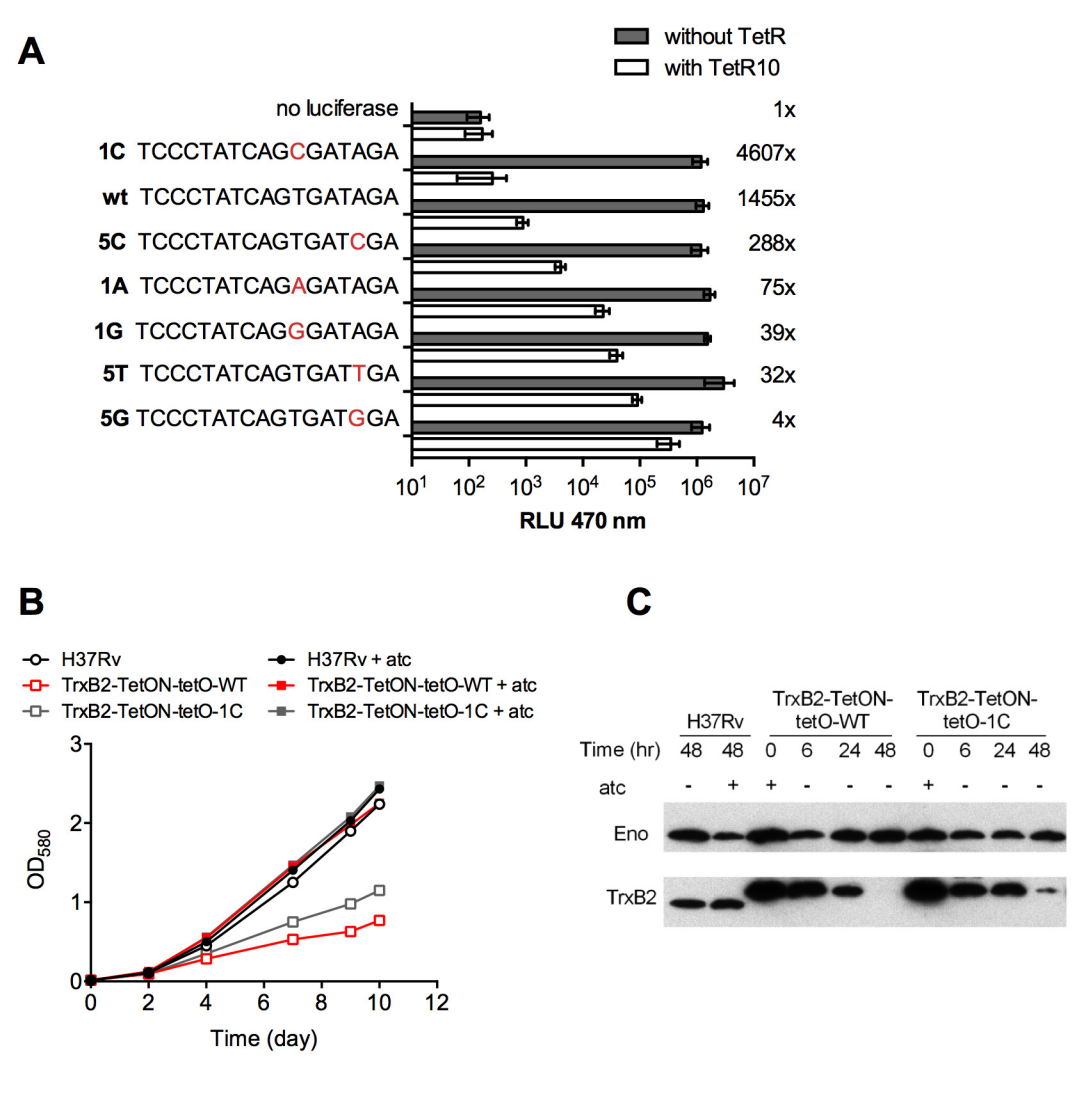
Design and characterization of leaky TrxB2-TetON mutants. (A) Repression of luciferase activity of leaky tet promoters by TetR in *M*. *smegmatis*. The x-axis specifies the promoter that was used to express luciferase and its tetO. Mutated nucleotides are shown in red. The *kanR* luciferase and *hygR* TetR plasmids were integrated into the *M*. *smegmatis* chromosome at the att-L5 and att-Tweety sites, respectively. Integers on the right indicate fold change in RLUs between bacteria without (gray bars) and with TetR (white bars). Data are means ± SD of eight replicates from at least two independent experiments. (B) Growth of H37Rv and TrxB2-TetON-tetO mutants in 7H9 medium in the presence or absence of atc. (C) Kinetics of TrxB2 depletion in TrxB2-TetON-tetO mutants in the absence of atc. TrxB2 in TrxB2-TetON-tetO mutants is of increased molecular weight due to the C-terminal DAS tag. Results in (B) and (C) are representative of three independent experiments.

Although purified TrxB2 has been shown to reduce H_2_O_2_ and other peroxides, little is known about the detoxification function of TrxB2 in a physiological setting [12,19]. Therefore, we sought to evaluate the impact of partial TrxB2 depletion on the susceptibility of *Mtb* to oxidative stress. Achieving partial TrxB2 depletion to an extent that does not affect viability but significantly reduces the intracellular TrxB2 protein amount is technically challenging with a DUC strain because of the steep atc dose response curve of this regulatory switch [22]. To circumvent this problem, we generated a panel of TrxB2-TetON mutants that contain point mutations in the operator of the tet promoter resulting in different degrees of constitutive, leaky transcription upon atc removal. Transcription from the mutated tet promoters is similar without TetR, however leaky repression results in a range of promoter activities without atc (Fig 3A). Two of the leaky TrxB2-TetON mutants, TrxB2-tetON-WT and TrxB2-tetON-1C, showed growth defects in the absence of atc (Fig 3B). Their growth defects correlated well with the protein depletion kinetics of TrxB2 (Fig 3C). These mutants thus achieved a phenotypically significant level of TrxB2 depletion yet retained enough TrxB2 to support growth. The moderate impact on growth of TrxB2-tetON-1C permitted the use of standard minimal inhibitory concentration (MIC) assays to measure how inhibition of TrxB2 affects susceptibility of *Mtb* to different chemical stresses.

**Fig 4:**
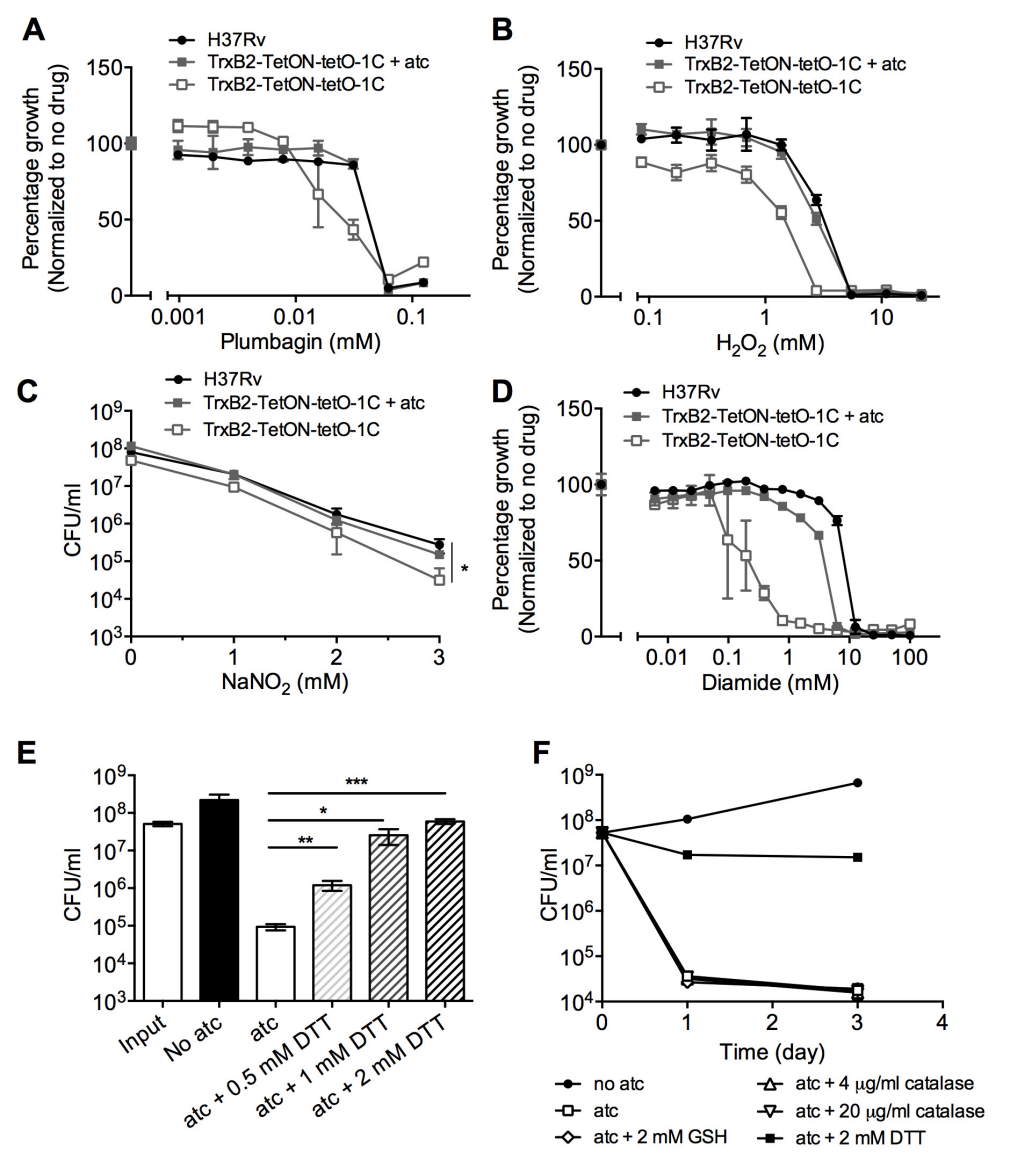
TrxB2 protects *Mtb* from thiol-specific oxidizing stress and contributes less to defense against oxidative and nitrosative stress. (A and B) Susceptibility of partially TrxB2-depleted *Mtb* to plumbagin (A) and H_2_O_2_ (B). TrxB2-TetON-tetO-1C was cultured in 7H9 medium without atc for 3 days to decrease TrxB2 expression before treatment with plumbagin and H_2_O_2_. OD_580_ was recorded and normalized to the corresponding strains without drug treatment. (C) Survival of *Mtb* strains after 4 days exposure to increasing concentrations of NaNO_2_ at pH 5.5. Data are means ± SD (n = 3 per group) and are representative of two independent experiments. (* p<0.05, one way ANOVA was used for group comparison). (D) Susceptibility of partially TrxB2-depleted *Mtb* to diamide. (E) Impact of dithiothreitol (DTT) on TrxB2 depletion-induced death in the TrxB2-DUC strain. *p<0.05, **p<0.01 and ***p<0.001 by unpaired Student’s t test. (F) Impact of extracellular glutathione (GSH) and catalase on TrxB2 depletion-induced death in the TrxB2-DUC strain. Data shown means ± SD (n = 3 per group) and are representative of two to three independent experiments.

Surprisingly, partial inhibition of TrxB2 did not affect *Mtb’s* susceptibility to growth inhibition or killing by plumbagin, a superoxide generator (Fig 4A and S7 Fig). TrxB2 silencing only caused a 2-fold shift of the MIC of H_2_O_2_, and we did not detect significant survival differences between wild type H37Rv and the TrxB2-tetON mutant following H_2_O_2_ exposure (Fig 4B and S7 Fig). Additionally, we measured *Mtb’s* susceptibility to reactive nitrogen species and found that TrxB2-silenced *Mtb* was only slightly less resistant to acidified nitrite at a high concentration (Fig 4C). In contrast, TrxB2-silenced *Mtb* was 8–16 fold more susceptible to growth inhibition by diamide, a thiol-specific oxidant (Fig 4D). This hypersusceptibility suggested a specific role for TrxB2 in detoxifying thiol-oxidizing stress.

To determine if thiol-specific oxidizing stress was responsible for the lethality caused by TrxB2 depletion, we tested if supplementation with the strong thiol-reducing agent dithiothreitol (DTT) could prevent death of TrxB2-depleted *Mtb*. Indeed, DTT rescued viability of TrxB2-DUC in a dose-dependent manner (Fig 4E). In contrast, neither glutathione nor catalase provided any survival benefit (Fig 4F). These results indicate that the primary function of TrxB2 in *Mtb* is to detoxify thiol-specific oxidative stress and that TrxB2 is the dominant thiol-reducing enzyme in *Mtb*.

### TrxB2 depletion perturbs growth-essential pathways

**Fig 5:**
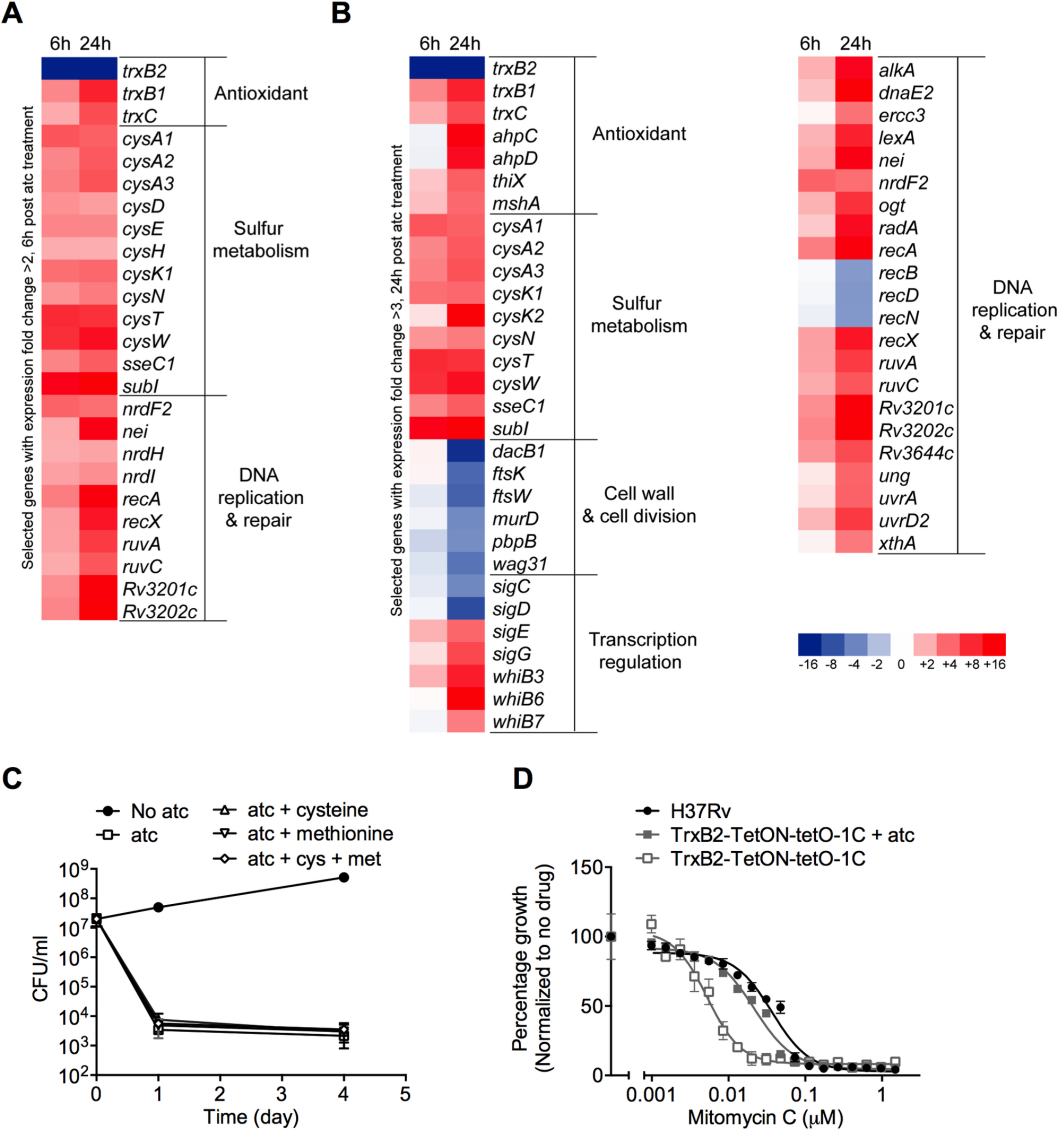
TrxB2 depletion perturbs growth-essential pathways. (A) Heat-map representation of selected genes with mean expression fold changes >2 in TrxB2-DUC at 6 h post atc treatment (adjusted p<0.02 by one-way ANOVA). (B) Heat-map representation of selected genes with mean expression fold change >3 in TrxB2-DUC at 24 h post atc treatment (adjusted p<0.02 by one-way ANOVA). (C) Impact of extracellular cysteine and methionine on TrxB2 depletion-induced death. Atc-treated TrxB2-DUC cultures were supplemented with 2 mM cysteine, 2 mM methionine or both. CFU were determined at the indicated time points. (D) Impact of partial TrxB2 depletion on susceptibility of *Mtb* to mitomycin C. TrxB2-TetON-tetO-1C was cultured in 7H9 medium without atc for 3 days to decrease TrxB2 expression before treatment with mitomycin C. Data in (C) and (D) are means ± SD (n = 3 per group) and are representative of three independent experiments.

We sought to investigate the pathways affected in TrxB2-depleted *Mtb* and analyzed the transcriptome changes associated with TrxB2 depletion. We found an early induction of 61 genes after 6 hours of atc treatment (fold change >2, p<0.02), 12 of which belong to sulfur metabolism pathways (Fig 5A). *Mtb* converts imported inorganic sulfate into adenosine 5’-phosposulfate (APS), which can be used for metabolite sulfation [25,26]. Alternatively, APS can be sequentially reduced for the biosynthesis of essential sulfur-containing metabolites, including cysteine, methionine and mycothiol. The first committed step in this reductive branch, the conversion of sulfate to sulfide by APS reductase (*cysH*), requires reducing potential supplied by the thioredoxin system [25,26]. We observed extensive up-regulation of sulfate importer genes (*cysT*, *cysW*, *cysA1* and *subI*) and genes in the reductive branch, including *cysH* and the O-acetylserine sulfhydrylase encoding *cysK1* and *cysK2*, which indicates a response to compensate for a defect in sulfur assimilation. Consistent with that, TrxB2 depletion also resulted in increased expression of *cysE*, encoding a serine acetyl transferase, which is required for *de novo* cysteine biosynthesis (Fig 5A). The expression of sulfur metabolism genes remained induced at 24 hrs post atc treatment (Fig 5B) and we asked whether death could be prevented or delayed by addition of reduced sulfur metabolites. A *cysH* deletion mutant was viable and had no growth defect, as long as it was supplemented with either 2 mM cysteine or methionine [27]. However, neither cysteine nor methionine protected TrxB2-depleted *Mtb* from death, indicating that TrxB2 is required for other essential pathways besides sulfur metabolism (Fig 5C).

Indeed, among the most highly up-regulated genes after 24 h of TrxB2 depletion were those involved in DNA metabolism (Fig 5B). We observed extensive up-regulation of genes involved in three DNA repair pathways, including base excision repair (*nei*, *alkA*, *ung*, *ogt* and *xthA*,), nucleotide excision repair (*ercc3*, *uvrA* and *uvrD2*), and homologous recombination (*recA*, *ruvA* and *ruvC*), suggesting that inhibition of TrxB2 was associated with DNA damaging stress. In support of this, we found that partial TrxB2 depletion decreased *Mtb’s* tolerance to genotoxic stress caused by mitomycin C, a potent DNA crosslinker (Fig 5D).

Of note, several genes involved in cell division were significantly down regulated in TrxB2-depleted *Mtb* (Fig 5B) consistent with the observed cell elongation (Fig 1I). The induction of antioxidant genes (*trxB1*, *trxC*, *thiX*, *ahpC*, *ahpD* and *mshA*) and *whiB3* encoding an intracellular redox sensor and regulator [28] further supports that TrxB2 depletion induces thiol-oxidizing stress.

Because DTT rescued survival of TrxB2-depleted *Mtb* (Fig 4E and 4F) we investigated its impact on the transcriptional changes caused by TrxB2 depletion. DTT treatment alleviated most of the mRNA changes associated with TrxB2 depletion without affecting atc-mediated transcriptional silencing and proteolytic degradation of TrxB2 (S8 and S9 Figs). It reduced the expression of most antioxidant genes to basal levels, suppressed the induction of sulfur metabolism genes, reduced suppression of cell division genes and decreased the activation of genes involved in DNA repair (S9 Fig).

Together, these data demonstrate that death following TrxB2 depletion was caused by pleiotropic effects on a number of growth-essential pathways, including sulfur and DNA metabolism, and was mediated primarily through exhaustion of thiol-reducing power (S10 Fig).

### Impact of partial TrxB2 depletion on susceptibility of *Mtb* to antimicrobial compounds

**Fig 6:**
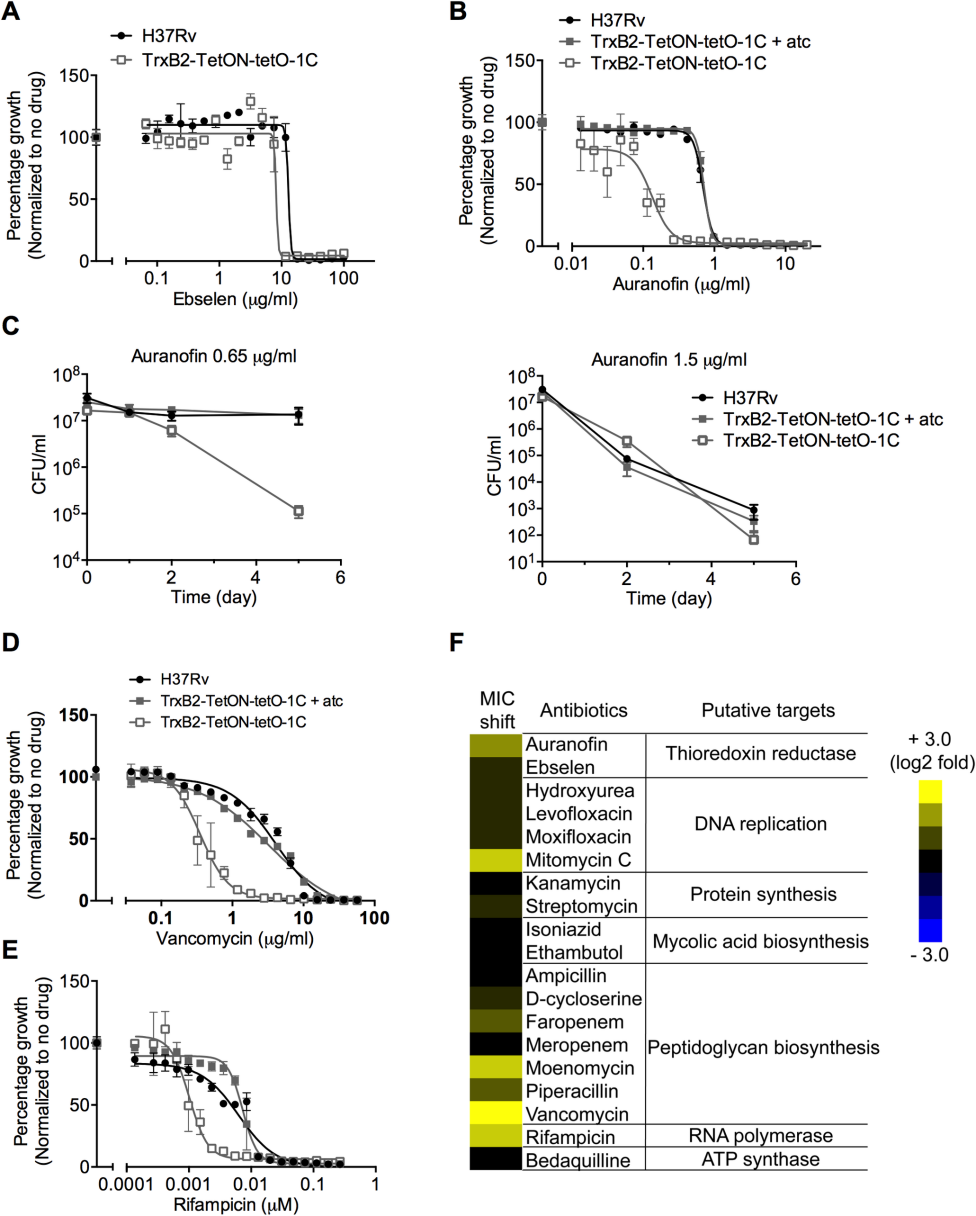
Impact of partial TrxB2 depletion on susceptibility of *Mtb* to antimicrobial compounds. (A and B) Impact of partial TrxB2 depletion on susceptibility of *Mtb* to TrxB2 inhibitors ebselen (A) and auranofin (B). TrxB2-TetON-tetO-1C was washed and suspended in 7H9 medium without atc, then cultured for 3 days to decrease TrxB2 expression before treatment with ebselen or auranofin. OD_580_ was recorded and normalized to the corresponding strain without drug treatment. (C) Survival of strains after exposure to 0.65 μg/ml or 1.5 μg/ml auranofin. (D and E) Impact of partial TrxB2 depletion on susceptibility of *Mtb* to vancomycin (D) and rifampicin (E). (F) Heat-map representation of MIC_90_ shift of partially TrxB2 depleted *Mtb* to antimicrobial compounds. The MIC_90_ shifts are shown as the ratio of the MIC_90_ for H37Rv to the MIC_90_ for TrxB2-TetON-tetO-1C in the absence of atc. Data in (A) to (E) are means ± SD of three replicates and are representative of three independent experiments. Data shown in (F) are representative of at least two independent experiments.

We utilized the leaky TrxB2 knockdown mutants to evaluate the specificity of two thioredoxin reductase inhibitors, ebselen and auranofin. Ebselen is a substrate of mammalian thioredoxin reductase, a competitive inhibitor of thioredoxin reductase from *E*. *coli*, and inhibits growth of *Mtb* [21]. *Mtb’s* susceptibility to ebselen was, however, not altered by partial TrxB2 depletion suggesting that ebselen inhibits *Mtb* growth by affecting other targets (Fig 6A). Auranofin, a gold-containing compound, was recently found to inhibit the enzymatic activity of *Mtb’s* TrxB2 *in vitro* and to kill *Mtb* [18]. Partial depletion of TrxB2 caused a 3.6-fold shift of the MIC of auranofin and sensitized *Mtb* to killing by 0.65 μg/ml auranofin, a concentration that did not affect viability of wt *Mtb* (Fig 6B and 6C). However, wt and mutant were killed similarly in the presence of a higher concentration of auranofin (Fig 6C). Our data suggest that TrxB2 is one of the major targets of auranofin, although auranofin likely inhibits multiple enzymes with reactive cysteine residues in *Mtb*, such as mycothione reductase [18].

To determine whether targeting TrxB2 sensitizes *Mtb* to other antimicrobial compounds, we screened the leaky TrxB2-TetON-1C mutant against a panel of antibiotics, including most of the first and second line anti-TB drugs. We found TrxB2-depleted *Mtb* highly susceptible to the cell wall biosynthesis inhibitors vancomycin and moenomycin (Fig 6D and 6F). Moenomycin directly inhibits bacterial peptidoglycan glycosyltransferases, while vancomycin can block both transglycosylation and transpeptidation by binding to the terminal D-Ala-D-Ala residues of the peptide stem [29]. Other inhibitors of peptidoglycan transpeptidation such as ampicillin, did not affect TrxB2-depleted *Mtb* more than wt *Mtb* (Fig 6F). Thus inhibiting TrxB2 may impair transglycosylation, which could contribute to the lysis phenotype we observed. Unexpectedly, depleting TrxB2 decreased the MIC of rifampicin by 5.6 fold, suggesting that a compound that inhibits TrxB2 may synergize with this important first line anti-TB drug (Fig 6E).

## Discussion

The paucity of targets that are both biologically validated and susceptible to inhibition by drug-like small molecules, i.e. “druggable”, is a major bottleneck in antimycobacterial drug development. *Mtb*’s thioredoxin reductase TrxB2 has recently been shown to be druggable, yet its biological evaluation has not advanced beyond the prediction of its essentiality for growth of *Mtb* on standard agar plates [18]. Auranofin inactivates thioredoxin reductase *in vitro* but has multiple targets in bacteria, including in *Mtb* [18,30]. It was thus unknown how the specific inhibition of TrxB2 would affect *Mtb* in different environments including those encountered during acute and chronic infections. We addressed these questions using genetic strategies and found that inactivating TrxB2 quickly eradicated *Mtb* during the acute and, importantly, the chronic phase of mouse infection, validating TrxB2 as a valuable target for therapeutic intervention. Deliberately leaky TrxB2 knockdown mutants revealed that a TrxB2 inhibitor may synergize with rifampicin. Treatment combinations of rifampicin and a TrxB2 inhibitor could thus reduce the required drug dosage and limit the frequency of resistant mutants as shown for the synergistic action of carbapenems and rifampicin [31]. We used a leaky TrxB2 mutant to determine the specificity of two TrxB2 inhibitors. The MIC of ebselen was not affected by partial TrxB2 depletion, suggesting that ebselen inhibits *Mtb* growth primarily through targets other than TrxB2. Ebselen has been shown to bind covalently to a cysteine residue located near the antigen 85 complex (Ag85C) active site and may thereby disrupt the biosynthesis of the mycobacterial cell envelope [32,33]. Auranofin was significantly more active against TrxB2-depleted *Mtb* than wild type indicating that it exerts its antimycobacterial activity at least partially through inhibiting TrxB2. However, auranofin exhibits a higher affinity for human thioredoxin reductase than for bacterial enzymes [34]. Furthermore, auranofin, an FDA-approved anti-rheumatic drug, has immunosuppressive activities by inhibiting NF-κB signaling and decreasing the production of nitric oxide and pro-inflammatory cytokines, which are critical for anti-TB immune responses [35,36]. It also has anti-tumor activity through inhibition of proteasome-associated deubiquitinases [37–39]. The catalytic mechanisms of mammalian and bacterial thioredoxin reductases are significantly different and the crystal structure of TrxB2 has been solved [6,20]. It should thus be possible to identify inhibitors that are more specific for TrxB2 than auranofin. We expect the leaky TetON mutants we constructed for this study to facilitate the identification of such inhibitors.

In addition to determining TrxB2’s value as a potential target for drug development we wanted to gain insights into the physiological functions of TrxB2, especially its role in detoxifying oxidative stress. TrxB2 expression is induced upon oxidative and nitrosative stress and purified TrxB2 can mediate the reduction of H_2_O_2_, peroxide, and dinitrobenzene [12,19]. However, TrxB2-depleted *Mtb* was hypersensitive specifically to thiol-oxidizing stress, but not to other types of oxidants, and the thiol reductant DTT prevented death caused by TrxB2 depletion. DTT did not promote growth of TrxB2-depleted *Mtb*, likely because DTT is very labile in neural aqueous solution and it is therefore difficult to maintain a constant concentration over time. Alternatively, TrxB2 has a function beyond its enzymatic activity, which is required for optimal growth and cannot be replaced by DTT. Notwithstanding, these results indicate that the primary function of TrxB2 in *Mtb* is to detoxify thiol-specific oxidative stress. Its potential role in defending against H_2_O_2_, superoxide and nitrosative stress is likely redundant with other antioxidant systems.

Mycothiol, a low-molecular-weight thiol present in millimolar quantities in mycobacterial cells, is thought to function as the mycobacterial substitute for glutathione and serve as the major redox buffer system in *Mtb* [10]. *Mtb* mycothiol-deficient mutants have a dramatically reduced intracellular thiol concentration, require catalase for optimal growth *in vitro* and exhibit increased sensitivity to oxidants. However, they are viable *in vitro* and only slightly attenuated in immunecompetent mice [15,40]. In contrast, TrxB2 depletion caused rapid lytic death even in the absence of exogenous oxidative stress and death was only prevented by DTT, but not catalase, cysteine and glutathione. Furthermore, TrxB2-depleted *Mtb* was unable to establish and maintain infection in mice. These phenotypic differences between mutants of the two major mycobacterial thiol-reducing systems emphasize that the TrxB2-dependent system provides the dominant thiol-reducing source to maintain thiol redox homeostasis. Recently, upregulation of thioredoxin genes in mycothiol deficient *Mtb* has been observed supporting that the thioredoxin system can restore mycothiol [11,41]. Some genes involved in DNA and sulfur metabolism were also differentially expressed in both mycothiol and ergothioneine deficient *Mtb* [11], however, the majority of these was down regulated, while we found them induced in response to TrxB2 depletion. Thus, while some relationships exist between ergothionine, mycothiol and the thioredoxin system, they represent to a large degree systems with distinct activities in maintaining redox balance.

Depriving thiol-reducing power via TrxB2 depletion affected numerous essential processes, including sulfur and DNA metabolism pathways. The conversion of sulfate to sulfide by APS reductase (CysH) requires reducing potential from the thioredoxin system, which may explain why TrxB2 depletion induced extensive up-regulation of the genes involved in cysteine biosynthesis [25,42]. TrxB2 depletion also strongly induced three different mycobacterial DNA repair pathways and consistent with this caused hypersusceptibility to the genotoxic drug mitomycin C. Ribonucleotide reductase (RNR) requires reducing power from the thioredoxin system to catalyze the reduction of NTP to dNTP [43], but TrxB2 depletion did not lead to increased sensitivity to the RNR inhibitor hydroxyurea. This is possibly due to the presence of both class I and class II RNRs in *Mtb* while hydroxyurea only inhibits class I RNR [44]. It is also possible that other DNA biosynthesis and repair enzymes rely on the thioredoxin system, a hypothesis we are currently investigating.

Surprisingly, we found that TrxB2 depletion lysed replicating *Mtb*. We observed significant cell elongation preceding lytic death consistent with the observed down-regulation of cell division genes. TrxB2-depleted *Mtb* was also highly susceptible to the peptidoglycan glycosyltransferases inhibitors moenomycin and vancomycin, but not to inhibitors of peptidoglycan transpeptidation, mycolic acid synthesis and arabinogalactan synthesis. We speculate that some enzymes or regulatory proteins involved in transglycosylation may depend on the thioredoxin system to maintain their intracellular redox states and function. Inactivation of TrxB2 may impair transglycosylation and thereby contribute to bacterial lysis. This observation also suggests a connection between redox-homeostasis and cell-envelope integrity in *Mtb*. We can therefore not exclude that TrxB2 depletion caused increased permeability to the sensitized compounds, although TrxB2-depletion did not cause susceptibility to all high molecular weight antibiotics.

In summary, this work identified TrxB2 as the dominant thiol-reducing enzyme in *Mtb* and refined understanding of its physiological roles in defending against thiol-oxidative stress and maintaining growth-essential pathways. Our results establish the importance of TrxB2 in *Mtb* pathogenesis and validate the enzyme as a drug target. The leaky TetON mutants we developed will facilitate target-based whole cell screens for the identification of TrxB2 inhibitors and can help maintaining on-target activity during drug development. We expect this strategy of partial transcriptional silencing to be widely applicable and to facilitate chemical-genetic interaction studies for other growth-essential proteins in *Mtb* and other pathogens.

## Materials and Methods

### Ethics statement

All animal experiments were performed following National Institutes of Health guidelines for housing and care of laboratory animals and performed in accordance with institutional regulations after protocol review and approval by the Institutional Animal Care and Use Committee of Weill Cornell Medical College (Protocol Number 0601-441A).

### Strains, media and culture conditions

Wild type *Mtb* (H37Rv) and its derivative strains were grown in Middlebrook 7H9 medium supplemented with 0.2% glycerol, 0.05% Tween-80, 0.5% BSA, 0.2% dextrose and 0.085% NaCl or on Middlebrook 7H10 agar containing OADC (Becton Dickinson and Company) and 0.5% glycerol. For growth of the TrxB2 leaky mutants, the above media were supplemented with 400 ng/ml anhydrotetracycline.

### Construction of mutant strains

To generate *Mtb trxB2*-DUC, we first transformed wild type *Mtb* H37Rv with an attL5-site integration plasmid expressing *trxB2* and *trxC* under the control of P750 promoter to obtain a merodiploid strain; *trxB2* and the first 4 bps of *trxC* (the OFR of *trxB2* overlaps with the first 4 bps of *trxC* ORF) were then deleted from the merodiploid strain by allelic exchange as previously described [45,46]. After confirming deletion of the native copy of *trxB2* by Southern blot, we performed replacement transformations of attL5 inserts to generate TrxB2-DUC [22]. In the TrxB2-DUC mutant, TrxB2 was expressed under the control of a TetOFF promoter and with a C-terminal DAS+4 tag. We also introduced a copy of *trxC* under the control of its native promoter to the attL5 site of TrxB2-DUC. The leaky TrxB2-TetON mutants were generated by replacement transformation of *Mtb* Δ*trxB2*::P750-*trxB2-trxC* with plasmids containing *trxB2* under the control of leaky tet promoters. A copy of *trxC* under the control of its native promoter was also introduced to the attL5 site of leaky TrxB2-TetON mutants.

### Essentiality test of TrxB2 and TrxC for *in vitro* growth

We transformed *Mtb ΔtrxB2*::P750-*trxB2*-*trxC* mutant with zeocin resistant plasmids expressing *trxB2* and *trxC*, *trxB2*, *trxC* or vector control. The transformants were selected on zeocin containing 7H10 agar. *ΔtrxC* was isolated from *Mtb ΔtrxB2*::P750-*trxB2*-*trxC* transformed with the plasmid expressing only *trxB2*.

### Survival of *Mtb* during nonreplicating conditions

The PBS starvation assay was set up as previously described [22]. Bacteria were grown in 7H9 medium to mid-log phase, washed three times with PBST, and suspended in PBST. After incubation for 10 d, atc was added to the cultures of TrxB2-DUC, and CFU were determined by plating on 7H10 plates.

### Immunoblotting analysis of cytosolic proteins

We prepared cell lysates from mid-log phase culture by bead-beating cell pellets in lysis buffer (50 mM Tris HCl pH 7.4, 150mM NaCl and 2mM EDTA) containing protease inhibitor cocktail (Roche). We then centrifuged the lysates at 13,000 rpm for 20 min and sterilized the supernatant by passing through 0.22 μm Spin-X filters (Costar). 30–60 μg total protein were separated by SDS–PAGE and transferred to nitrocellulose membranes for probing with rabbit antisera against *Mtb* TrxB2, enolase (Eno), proteasome beta subunit (PrcB) and dihydrolipoamide acyltransferase (DlaT). Recombinant full-length Eno and TrxB2 were expressed with a C-terminal His tag, purified and used as antigen for immunization of rabbits.

### Analysis of bacterial lysis by immunoblotting

Culture filtrates were prepared as follows. *Mtb* strains were grown in 7H9 medium with 0.2% glycerol, 0.05% Tween-80, 0.5% BSA, 0.2% dextrose and 0.085% NaCl until the culture reached an OD of 0.6 ~ 0.8. Cultures were then washed three times with PBS to remove BSA and Tween-80. We next suspended the pellet in 7H9 medium supplemented with 0.2% glycerol, 0.2% dextrose and 0.085% NaCl. After incubation, culture supernatant was harvested by centrifugation and filtration through 0.22 μm filters. Filtrates were concentrated 100-fold by using 3K centrifugal filter units (Millipore) and analyzed by immunoblotting with antisera against DlaT, Eno, PcrB and Ag85B (Abcam, ab43019).

### Mouse infections

We infected female C57BL/6 mice (Jackson Laboratory) using an inhalation exposure system (Glas-Col) with mid-log phase *Mtb* to deliver approximately 200 bacilli per mouse. Mice received doxycycline containing mouse chow (2,000 ppm; Research Diets) starting at the indicated time-points. Lungs and spleens were homogenized in PBS, serially diluted and plated on 7H10 agar to quantify CFU. Upper left lung lobes were fixed in 10% buffered formalin, embedded in paraffin and stained with hematoxylin and eosin.

### Gene expression analysis by microarray

For transcriptome analysis of TrxB2-depleted *Mtb*, we grew TrxB2-DUC in 7H9 medium to an OD of 0.5~0.6 and then added 400 ng/ml atc. Samples were collected at 6 hr and 24 hr later. Each experiment was performed with at least three independent cultures. To determine the impact of DTT, TrxB2-DUC was treated with atc, DTT (2 mM) or both for 24 hrs. Microarray analysis was performed as previously described [47]. Cultures were mixed at a 1:1 ratio with GTC buffer containing guanidinium thiocyanate (4 M), sodium lauryl sulfate (0.5%), trisodium citrate (25 mM), and 2-mercaptoethanol (0.1 M) and pelleted by centrifugation. Bacterial RNA was isolated and labeled using a Low Input Quick Amp Labeling Kit (Agilent) according to the manufacturer’s instruction. Custom-designed *Mtb* H37Rv whole genome microarray (GEO platform GPL16177) were used. Analysis and clustering were performed with Agilent GeneSpring software. One-way ANOVA was used to compare microarray data, with Benjamini–Hochberg correction for multiple hypothesis testing. All the data have been deposited in the GEO database with the accession numbers GSE72328, GSE72329, GSE72330 and GSE78894.

### Stress assays

For oxidative stress, *Mtb* strains were grown to mid-log phase and washed twice in 7H9 medium. Bacterial single cell suspension was then prepared by centrifuging the cultures at 800 g for 10 min to remove clumps. We then adjusted the OD to 0.03, treated *Mtb* strains with H_2_O_2,_ plumbagin, diamide or acidified nitrite and determined CFU by plating.

### Antibiotic sensitivity assays

*Mtb* was grown to mid-log phase and diluted to an OD of 0.03 in 7H9 medium. Bacteria were then exposed to 1.5-fold serial dilution of antimicrobial compounds. Optical density was recorded after 14 days and normalized to the corresponding strains without drug treatment. Minimum inhibitory concentration is defined as the lowest concentration of a drug at which bacterial growth was inhibited at least 90%, as compared to the control containing no antimicrobial compounds. Ampicillin, auranofin, D-cycloserine, ebselen, ethambutol, faropenem, hydroxyurea, isoniazid, kanamycin, levofloxacin, meropenem, mitomycin C, moxifloxacin, piperacillin, rifampicin, streptomycin and vancomycin were purchased from Sigma Aldrich, St. Louis, MO. Moenomycin was from Santa Cruz Biotechnology. Bedaquilline was received as a gift from C. Barry.

### Statistical analysis

One-way ANOVA was used for multiple group comparisons. Two-tailed unpaired Student’s t test was used for the analysis of differences between two groups. Statistical significance was defined as P < 0.05 unless otherwise stated. No statistical methods were used to predetermine sample size.

## Supporting Information

S1 Fig TrxB2 is required for growth of *Mtb*. (A) Map of the *trxB2-trxC* genomic region in H37Rv and Δ*trxB2*::P750-*trxB2-trxC*. To construct Δ*trxB2*::P750-*trxB2-trxC*, we first generated a merodiploid strain by integrating a second copy of the *trxB2*-*trxC* operon into the attL5 site. Then *trxB2* and the first 4 bps of *trxC*, which overlap with *trxB2*, were replaced with a hygromycin cassette by homologous recombination. Inactivated *trxC* lacking the first 4 bps is marked with an asterisk. (B and C) Southern blot of *Xba*I-digested genomic DNA from H37Rv and seven Δ*trxB2*::P750-*trxB2-trxC* candidates probed with probes 1 (B) and 2 (C) as indicated in (A). (D) To test essentiality of *trxB2* and *trxC*, Δ*trxB2*::P750-*trxB2-trxC* was transformed with integrative plasmids expressing *trxB2*-*trxC*, *trxB2*, *trxC* or vector control to replace the *trxB2-trxC* copy in the attL5 site. Only plasmids containing *trxB2* yielded colonies demonstrating that *trxB2* but not *trxC* is required for growth.
(PDF)

S2 Fig Construction of TrxB2-DUC. To generate the dual control (DUC) mutant, the *trxB2*-*trxC* plasmid located in the attL5 site of Δ*trxB2*::P750-*trxB2*-*trxC* was replaced with a plasmid containing DAS-tagged *trxB2* expressed from the tet-operator containing promoter P750, *trxC* with its native promoter, and reverse tet repressor with a constitutive promoter. In addition the mutant was transformed with a plasmid that integrates in the tweety phage attachment site and expresses the SspB adaptor protein under control of wt tet repressor.
(PDF)

S3 Fig Semiquantitative immunoblot analysis of TrxB2 in TrxB2-DUC. Immunoblot of protein extracts from TrxB2-DUC treated with atc for 6, 24 and 48 hrs. Serially diluted H37Rv lysate was used to determine the limit of detection of TrxB2. Eno serves as loading control.
(PDF)

S4 Fig NadE depletion does not cause detectable lysis of *Mtb*. Immunoblot analysis of DlaT, Eno, and Ag85B from culture filtrates of H37Rv, TrxB2-DUC and NadE-DUC treated or not with atc for 6 days.
(PDF)

S5 Fig TrxB2 depletion in *Mtb* causes cell elongation. Representative images of H37Rv and TrxB2-DUC treated or not with atc for 4 days. Samples were examined with bright-field microscopy. Data processing was performed with ImageJ.
(PDF)

S6 Fig Silencing TrxB2 during the acute phase of infection prevented pulmonary pathology in mice. Gross pathology (A) and H&E staining (B) of lung tissue sections from infected mice receiving doxy-containing food starting from day 0, day 10 or not treated. Lungs were isolated on day 35 and 56 post-infection. Scale bar, 1.0 mm.
(PDF)

S7 Fig Susceptibility of partially TrxB2-depleted *Mtb* to oxidative stress. (A) Immunoblot analysis of TrxB2 in protein extracts prepared from cultures used in (B). (B) TrxB2-TetON-tetO-1C mutant was cultured in the absence of atc for 3 days to decrease TrxB2 expression. *Mtb* strains were then exposed to 0.25 mM plumbagin for 5 h, to 5.4 mM H_2_O_2_ for 4 h or to 50 mM diamide for 8 h and bacterial survival was determined by CFU. * p<0.05, ** p<0.01, one way ANOVA was used for group comparison. Results are representative of three independent experiments.
(PDF)

S8 Fig DTT treatment does not affect atc-mediated TrxB2 depletion in TrxB2-DUC. Immunoblot of protein extracts from TrxB2-DUC with different treatment as indicated. Blot was probed with TrxB2-specific and Eno-specific (loading control) antisera.
(PDF)

S9 Fig DTT mitigates the transcriptional impact of TrxB2 depletion. Heat-map representation of expression level of fold-changes of selected genes in response to atc and DTT treatment. mRNA abundances in TrxB2-DUC treated with atc, DTT or both were compared to those in untreated TrxB2-DUC. One-way ANOVA was used for group comparison (n = 3 per group), with Benjamini–Hochberg correction for multiple hypothesis testing. Selected genes with mean expression fold change >3 at 24 h post atc treatment are shown (adjusted p<0.02).
(PDF)

S10 Fig Model for the activities of thioredoxin reductase in *Mtb*. *Mtb’s* thioredoxin system is composed of thioredoxin reductase (encoded by *trxB2*), thioredoxin (Trx) and NADPH. TrxB2 catalyzes the disulfide-thiol exchange of thioredoxins and thioredoxin-like proteins using electrons from NADPH. Thioredoxins and thioredoxin-like proteins are then able to reduce their substrates and maintain essential biological pathways, such as DNA replication, genome integrity, sulfur metabolism and cell wall processes. Loss of TrxB2 activity results in thiol-oxidizing stress, which damages DNA, perturbs sulfur metabolism, affects cell wall processes and leads to lysis of *Mtb*.
(PDF)
