# Supplementary figures and images for: Mycobacterium tuberculosis Thioredoxin Reductase Is Essential for Thiol Redox Homeostasis but Plays a Minor Role in Antioxidant Defense
Source: PLoS Pathog. 2016 Jun 1;12(6):e1005675. doi: 10.1371/journal.ppat.1005675 (PMC4889078; doi:10.1371/journal.ppat.1005675)

**A**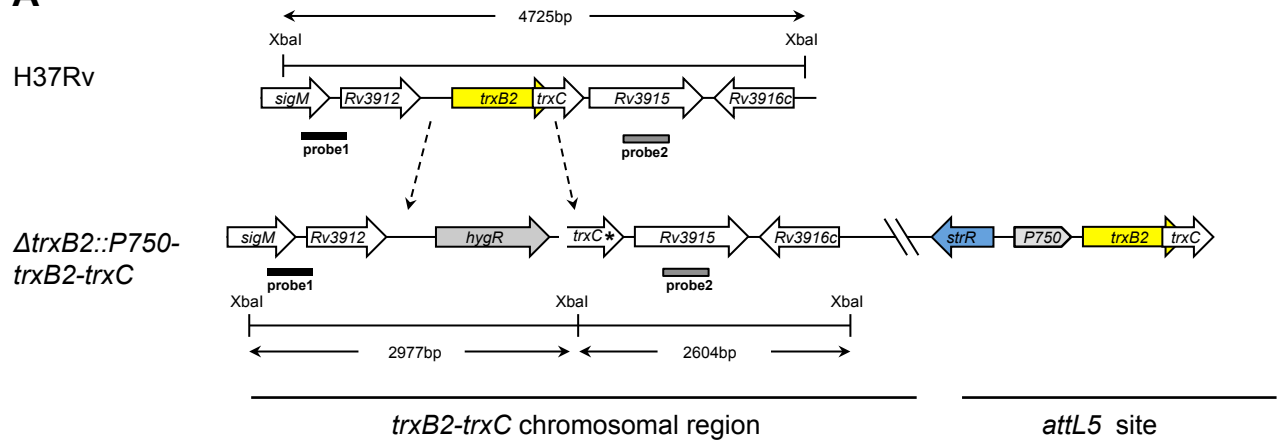**B**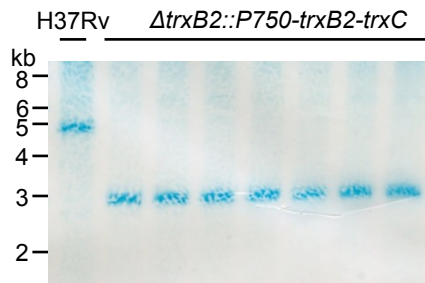**C**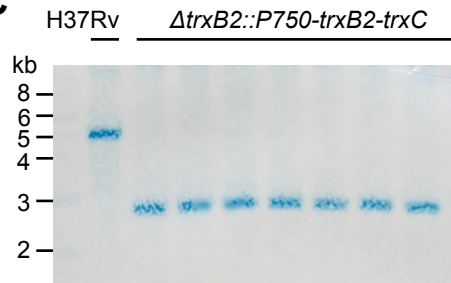**D**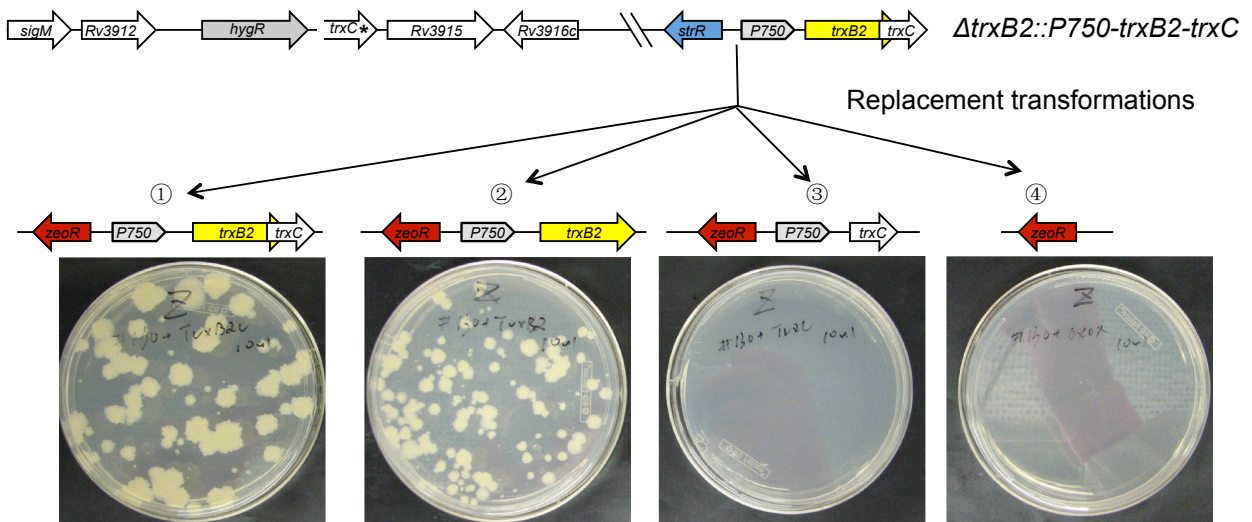

Supplement: S1 Fig — (A) Map of the trxB2-trxC genomic region in H37Rv and ΔtrxB2::P750-trxB2-trxC. To construct ΔtrxB2::P750-trxB2-trxC, we first generated a merodiploid strain by integrating a second copy of the trxB2-trxC operon into the attL5 site. Then trxB2 and the first 4 bps of trxC, which overlap with trxB2, were replaced with a hygromycin cassette by homologous recombination. Inactivated trxC lacking the first 4 bps is marked with an asterisk. (B and C) Southern blot of XbaI-digested genomic DNA from H37Rv and seven ΔtrxB2::P750-trxB2-trxC candidates probed with probes 1 (B) and 2 (C) as indicated in (A). (D) To test essentiality of trxB2 and trxC, ΔtrxB2::P750-trxB2-trxC was transformed with integrative plasmids expressing trxB2-trxC, trxB2, trxC or vector control to replace the trxB2-trxC copy in the attL5 site. Only plasmids containing trxB2 yielded colonies demonstrating that trxB2 but not trxC is required for growth. (PDF) [file ppat.1005675.s001.pdf]

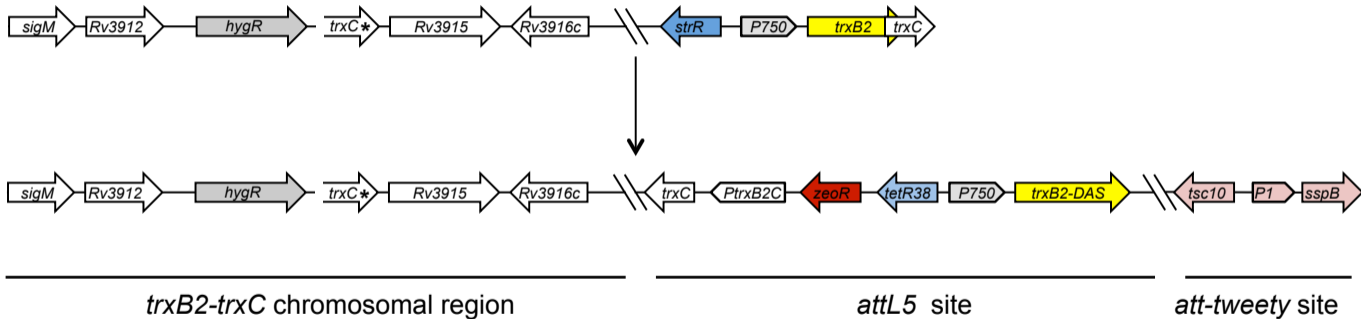

Supplement: S2 Fig — To generate the dual control (DUC) mutant, the trxB2-trxC plasmid located in the attL5 site of ΔtrxB2::P750-trxB2-trxC was replaced with a plasmid containing DAS-tagged trxB2 expressed from the tet-operator containing promoter P750, trxC with its native promoter, and reverse tet repressor with a constitutive promoter. In addition the mutant was transformed with a plasmid that integrates in the tweety phage attachment site and expresses the SspB adaptor protein under control of wt tet repressor. (PDF) [file ppat.1005675.s002.pdf]

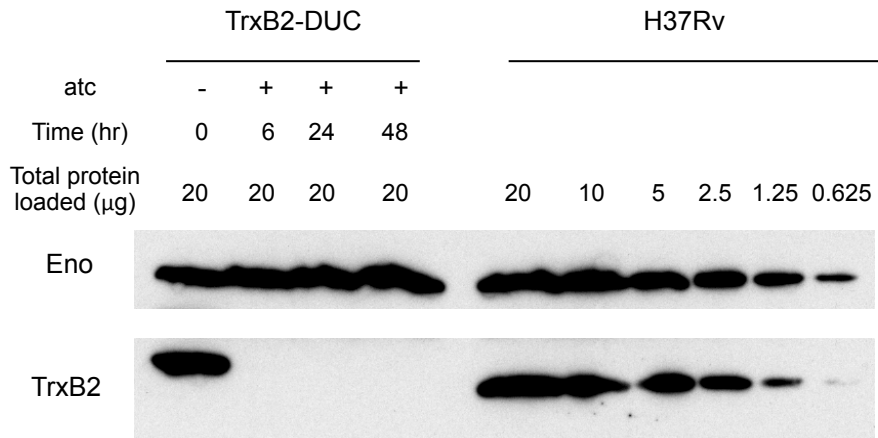

Supplement: S3 Fig — Immunoblot of protein extracts from TrxB2-DUC treated with atc for 6, 24 and 48 hrs. Serially diluted H37Rv lysate was used to determine the limit of detection of TrxB2. Eno serves as loading control. (PDF) [file ppat.1005675.s003.pdf]

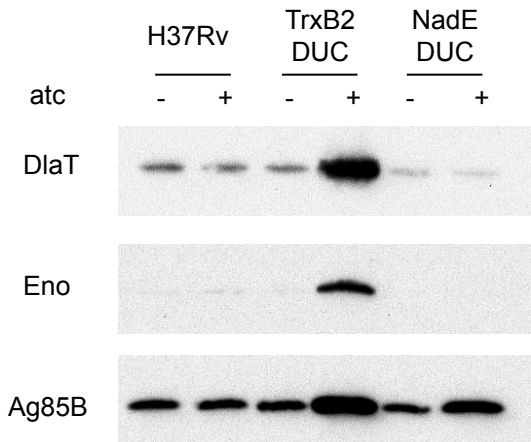

Supplement: S4 Fig — Immunoblot analysis of DlaT, Eno, and Ag85B from culture filtrates of H37Rv, TrxB2-DUC and NadE-DUC treated or not with atc for 6 days. (PDF) [file ppat.1005675.s004.pdf]

H37Rv

TrxB2-DUC

- ATC

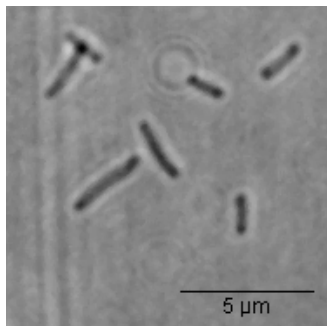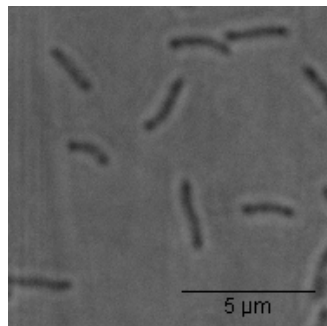

+ ATC

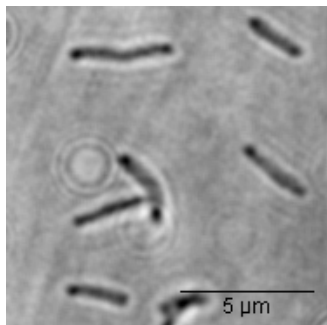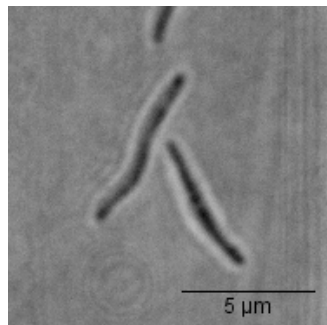

Supplement: S5 Fig — Representative images of H37Rv and TrxB2-DUC treated or not with atc for 4 days. Samples were examined with bright-field microscopy. Data processing was performed with ImageJ. (PDF) [file ppat.1005675.s005.pdf]

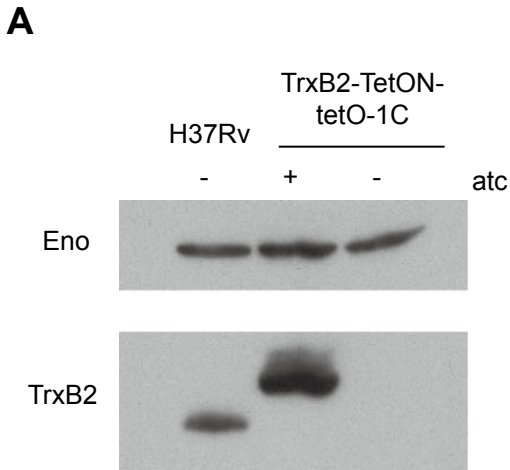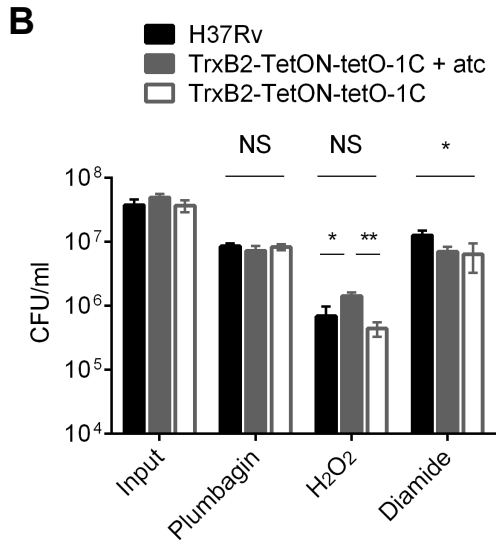

Supplement: S7 Fig — (A) Immunoblot analysis of TrxB2 in protein extracts prepared from cultures used in (B). (B) TrxB2-TetON-tetO-1C mutant was cultured in the absence of atc for 3 days to decrease TrxB2 expression. Mtb strains were then exposed to 0.25 mM plumbagin for 5 h, to 5.4 mM H2O2 for 4 h or to 50 mM diamide for 8 h and bacterial survival was determined by CFU. * p<0.05, ** p<0.01, one way ANOVA was used for group comparison. Results are representative of three independent experiments. (PDF) [file ppat.1005675.s007.pdf]

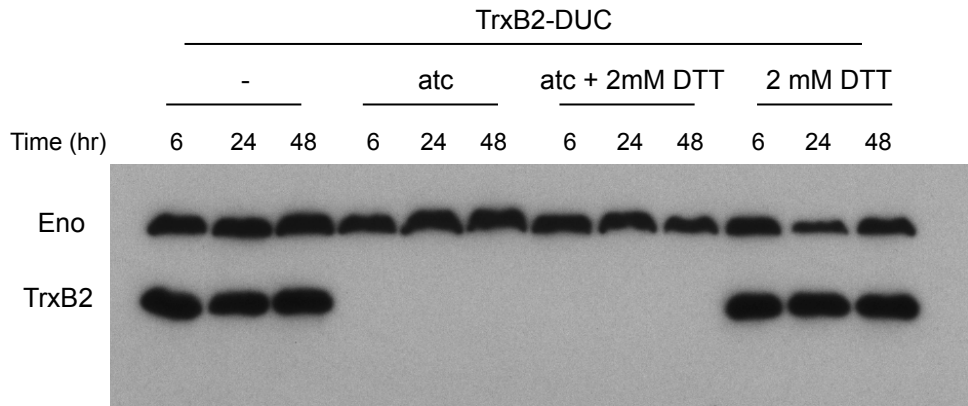

Supplement: S8 Fig — Immunoblot of protein extracts from TrxB2-DUC with different treatment as indicated. Blot was probed with TrxB2-specific and Eno-specific (loading control) antisera. (PDF) [file ppat.1005675.s008.pdf]

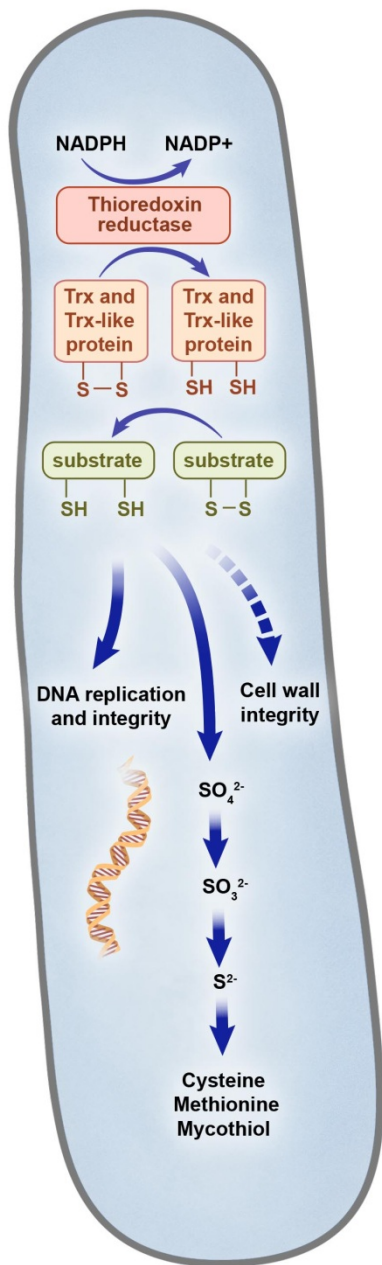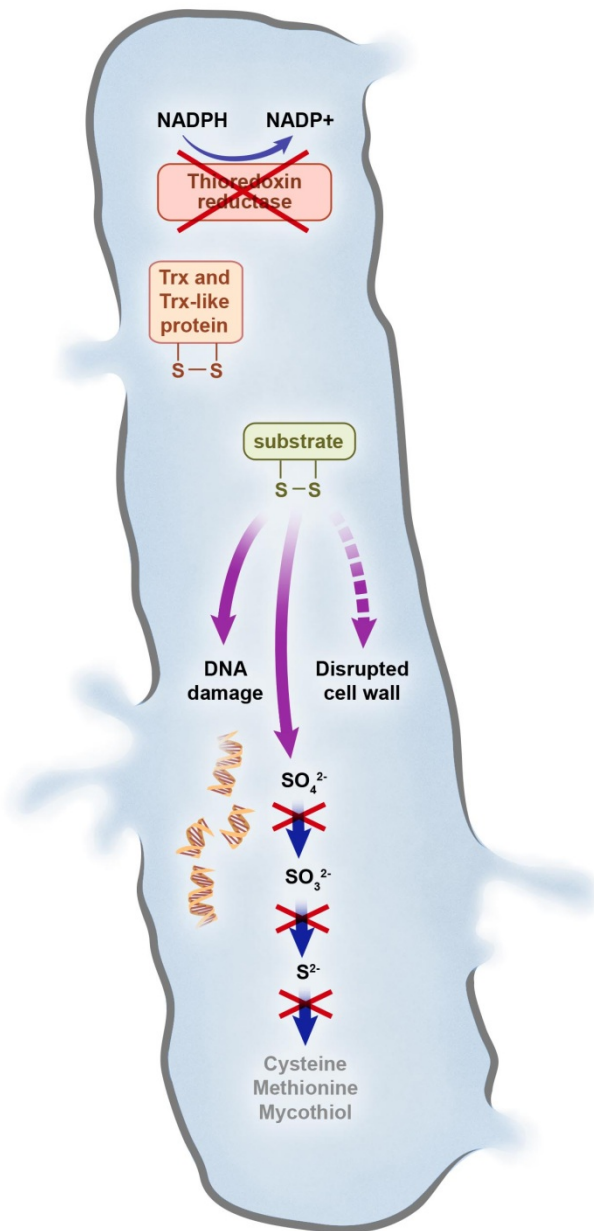

Supplement: S10 Fig — Mtb’s thioredoxin system is composed of thioredoxin reductase (encoded by trxB2), thioredoxin (Trx) and NADPH. TrxB2 catalyzes the disulfide-thiol exchange of thioredoxins and thioredoxin-like proteins using electrons from NADPH. Thioredoxins and thioredoxin-like proteins are then able to reduce their substrates and maintain essential biological pathways, such as DNA replication, genome integrity, sulfur metabolism and cell wall processes. Loss of TrxB2 activity results in thiol-oxidizing stress, which damages DNA, perturbs sulfur metabolism, affects cell wall processes and leads to lysis of Mtb. (PDF) [file ppat.1005675.s010.pdf]
