# Supplementary material for: Mycobacterium tuberculosis Thioredoxin Reductase Is Essential for Thiol Redox Homeostasis but Plays a Minor Role in Antioxidant Defense
Source: PLoS Pathog. 2016 Jun 1;12(6):e1005675. doi: 10.1371/journal.ppat.1005675 (PMC4889078; doi:10.1371/journal.ppat.1005675)
Supplement: S6 Fig — Gross pathology (A) and H&E staining (B) of lung tissue sections from infected mice receiving doxy-containing food starting from day 0, day 10 or not treated. Lungs were isolated on day 35 and 56 post-infection. Scale bar, 1.0 mm. (PDF) [file ppat.1005675.s006.pdf]

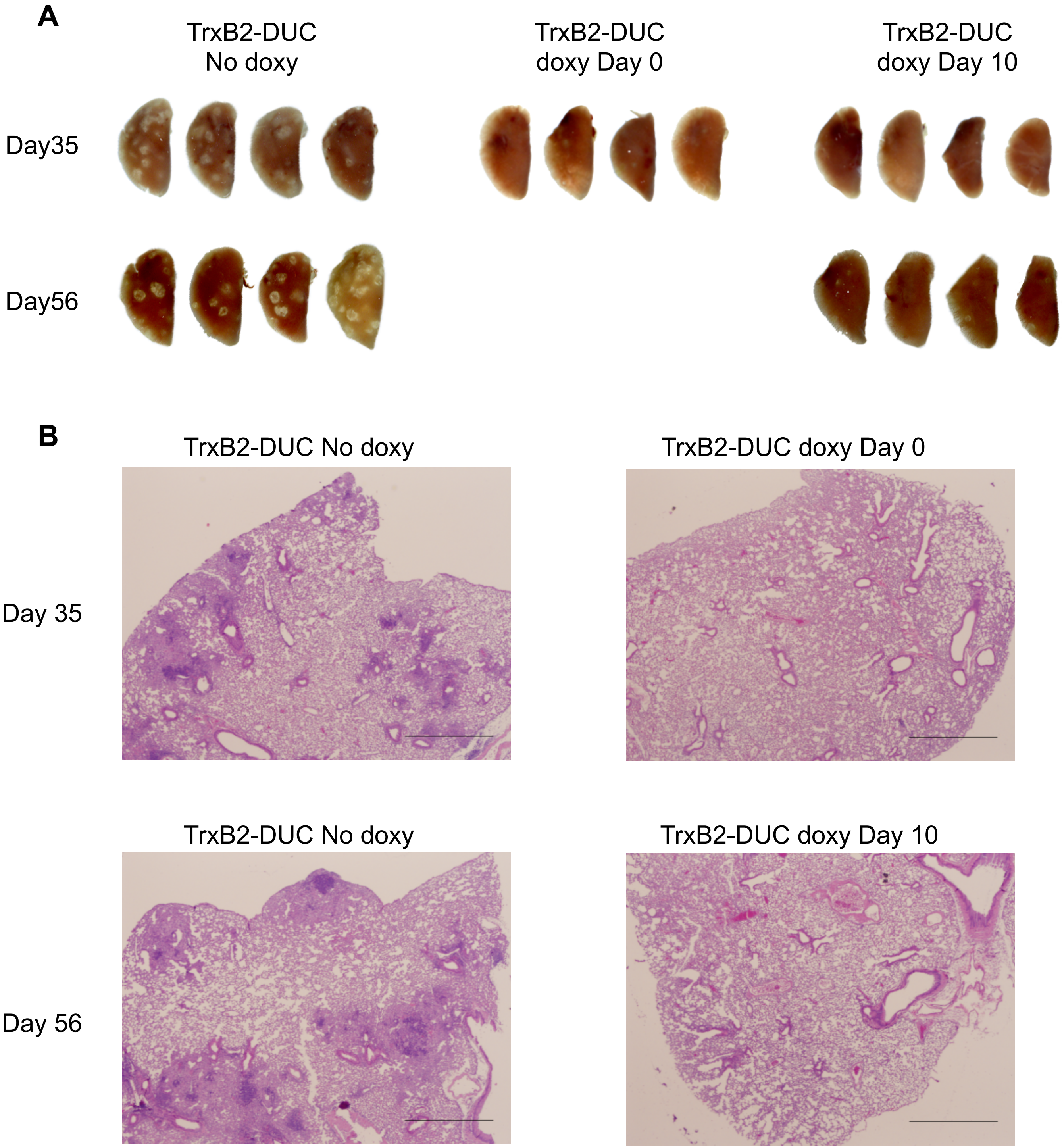
A
TrxB2-DUC
No doxy
TrxB2-DUC
doxy Day 0
TrxB2-DUC
doxy Day 10
Day35
Day56
B
TrxB2-DUC No doxy
TrxB2-DUC doxy Day 0
Day 35
TrxB2-DUC No doxy
TrxB2-DUC doxy Day 10
Day 56
