# Supplementary material for: Mycobacterium tuberculosis Thioredoxin Reductase Is Essential for Thiol Redox Homeostasis but Plays a Minor Role in Antioxidant Defense
Source: PLoS Pathog. 2016 Jun 1;12(6):e1005675. doi: 10.1371/journal.ppat.1005675 (PMC4889078; doi:10.1371/journal.ppat.1005675)
Supplement: S9 Fig — Heat-map representation of expression level of fold-changes of selected genes in response to atc and DTT treatment. mRNA abundances in TrxB2-DUC treated with atc, DTT or both were compared to those in untreated TrxB2-DUC. One-way ANOVA was used for group comparison (n = 3 per group), with Benjamini–Hochberg correction for multiple hypothesis testing. Selected genes with mean expression fold change >3 at 24 h post atc treatment are shown (adjusted p<0.02). (PDF) [file ppat.1005675.s009.pdf]

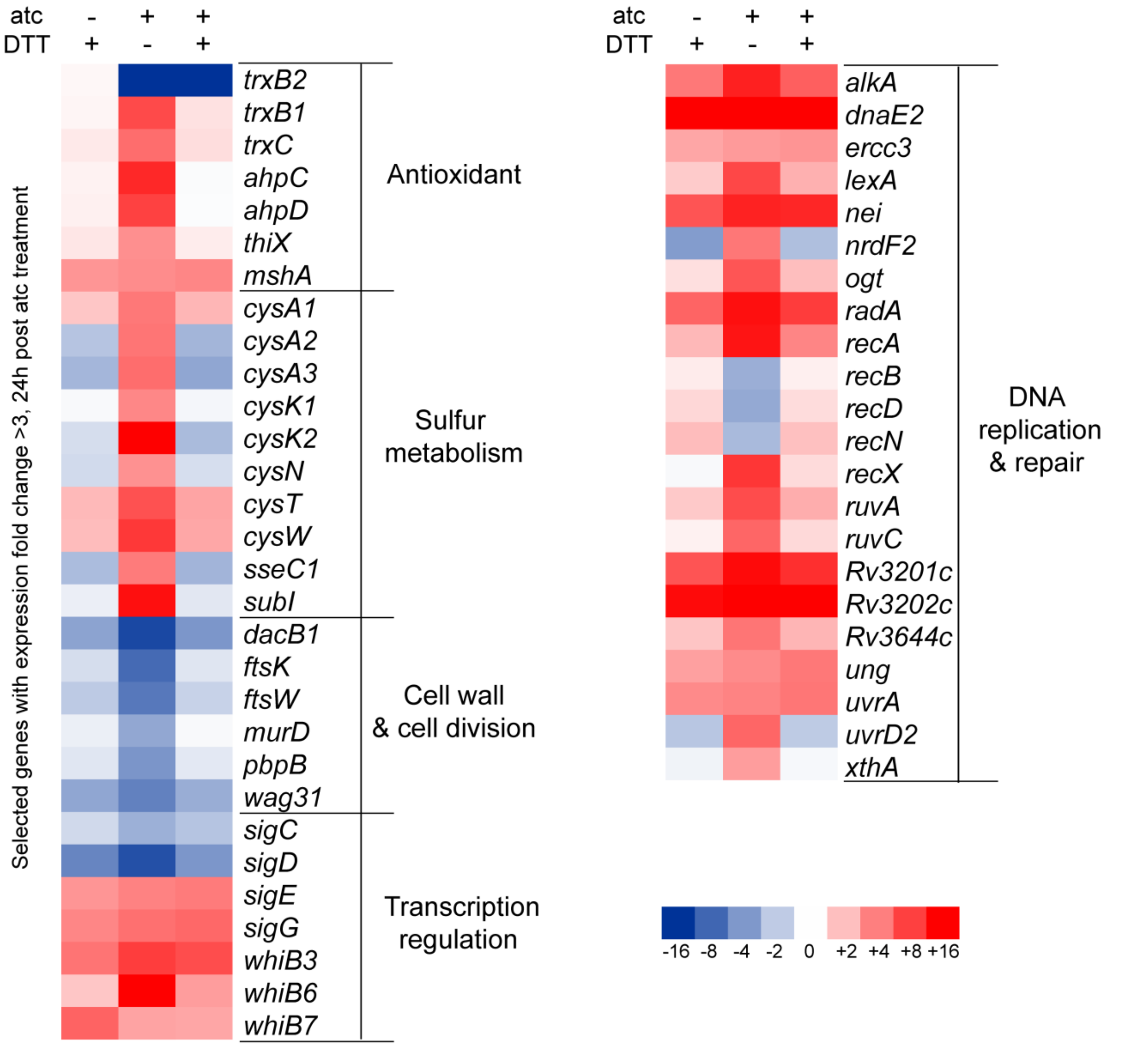
atc - + +
DTT + - +
Selected genes with expression fold change >3, 24h post atc treatment
trxB2
trxB1
trxC
ahpC
ahpD
thiX
mshA
Antioxidant
cysA1
cysA2
cysA3
cysK1
cysK2
cysN
cysT
cysW
sseC1
subI
Sulfur metabolism
dacB1
ftsK
ftsW
murD
pbpB
wag31
Cell wall & cell division
sigC
sigD
sigE
sigG
whiB3
whiB6
whiB7
Transcription regulation
atc - + +
DTT + - +
alkA
dnaE2
ercc3
lexA
nei
nrdF2
ogt
radA
recA
recB
recD
recN
recX
ruvA
ruvC
Rv3201c
Rv3202c
Rv3644c
ung
uvrA
uvrD2
xthA
DNA replication & repair
-16 -8 -4 -2 0 +2 +4 +8 +16
